# Platinum-Promoted Ga/Al_2_O_3_ as Highly Active, Selective, and Stable Catalyst for the Dehydrogenation of Propane**

**DOI:** 10.1002/anie.201404460

**Published:** 2014-07-02

**Authors:** Jesper J H B Sattler, Ines D Gonzalez-Jimenez, Lin Luo, Brien A Stears, Andrzej Malek, David G Barton, Beata A Kilos, Mark P Kaminsky, Tiny W G M Verhoeven, Eline J Koers, Marc Baldus, Bert M Weckhuysen

**Affiliations:** Inorganic Chemistry and Catalysis Group, Debye Institute for Nanomaterials Science, Utrecht UniversityUniversiteitsweg 99, 3584 CG Utrecht (The Netherlands); Hydrocarbons R&D, The Dow Chemical Company2301 N. Brazosport Blvd., Freeport, TX 77541 (USA); Core R&D, The Dow Chemical Company1776 Building, Midland, MI 48674 (USA); NMR Spectroscopy Research Group, Bijvoet Centre for Biomolecular Research Utrecht UniversityPadualaan 8, 3584 CH Utrecht (The Netherlands); Eindhoven University of TechnologyP.O. Box 513, 5600 MB Eindhoven (The Netherlands)

**Keywords:** gallium oxide, heterogeneous catalysis, platinum, propane dehydrogenation, synergistic effects

## Abstract

A novel catalyst material for the selective dehydrogenation of propane is presented. The catalyst consists of 1000 ppm Pt, 3 wt % Ga, and 0.25 wt % K supported on alumina. We observed a synergy between Ga and Pt, resulting in a highly active and stable catalyst. Additionally, we propose a bifunctional active phase, in which coordinately unsaturated Ga^3+^ species are the active species and where Pt functions as a promoter.

The recent exploration and production of hydrocarbons from shale basins in the USA such as in Barnett, Marcellus, Haynesville, and Eagle Ford, has led to a rebound in its energy competitiveness. Currently, the USA is at the lowest level of crude oil imports in 25 years.[1] While oil production has greatly increased in these shale plays, natural gas has increased even more significantly, with the Energy Information Agency projecting that by 2040, 50 % of the natural gas production within the USA will come from shale.[2] This new source of hydrocarbons has the potential to impact the worldwide supply of natural gas, because shale formations are found throughout the world. A recent study estimates that there are 207 trillion cubic meters of technically recoverable shale gas globally. China is estimated to have the world’s largest reserves in shale at 32 trillion cubic meters.[3] Although most shale gas outside of the USA is not currently produced, it is reasonable to expect that these low-cost feedstocks for chemicals and fuels production will become available worldwide. These developments will without a doubt impose significant technical and economic challenges and opportunities on the chemical industry as a whole.

Since substantial amounts of heavier paraffins, such as propane are obtained from shale gas deposits, there is a vast and growing interest in utilizing propane dehydrogenation (PDH) technologies for the on purpose production of propene.[4] Within this context, it is important to mention that there have been five newly announced PDH units in the USA, while 9 to 17 PDH units may be built in China.[5] The majority of these projects is based on one of the two primary existing technologies for PDH; i.e., the Oleflex process from UOP and the CATOFIN process from CB&I Lummus.[6] Although substantial improvements in catalyst materials (Pt-Sn/Al_2_O_3_ for Oleflex and Cr/Al_2_O_3_ for CATOFIN) and process conditions have been made for both technologies, challenges related to their activity, stability, and selectivity still have to be overcome.

Here we present a new family of very stable, active, and selective catalyst materials for the dehydrogenation of propane to propene based on Pt–Ga/Al_2_O_3_. A few papers have already been published, in which Pt and Ga were combined to produce PDH catalysts, but in these systems Ga is deemed to function as a promoter element, with Pt being the active dehydrogenation element.[7] This is in contrast with our current catalyst, in which Pt is present in minute amounts and Ga is the active dehydrogenation element. A clear synergistic effect is observed between both components, which results in a very stable catalyst material that is highly resistant to deactivation, e.g., by coking.

To perform a systematic study, a series of nine catalyst materials was prepared by depositing 1000 ppm Pt, 1.5 or 3 wt % Ga, and 0.25 wt % K on an alumina support. Details of the catalyst characterization (X-ray diffraction (XRD), transmission electron microscopy energy-dispersive X-ray spectroscopy (TEM-EDX), and N_2_-physisorption) are given in the Supporting Information (Figures S1 and S2, and Table S1). From these characterization data it is concluded that all the compounds are homogeneously and highly dispersed on the catalyst materials as there is no evidence for the presence of crystalline nanoparticles.

The catalytic performance of the prepared materials has been tested in a lab-scale reactor for eight successive dehydrogenation–regeneration cycles. Each cycle consists of a 15 min PDH step at 620 °C, followed by a treatment in air at 750 °C for 30 min. The reactor is flushed with He between these steps. The resultant gas stream is analyzed by gas chromatography as described in the Supporting Information (Figure S3). The setup also allows for the use of operando Raman and UV/Vis spectroscopy to track the deposition of coke on the catalyst materials.[8] A complete list of the catalysts prepared and their respective activity and selectivity data for the first, second, and eighth PDH cycle is summarized in Table 1. Additionally, Figure 1 shows a comparison between the conversion and selectivity obtained during the first eight cycles for the bare Al_2_O_3_ support, Pt, 3Ga, Pt3Ga, and Pt3GaK catalyst materials.

**Figure 1:**
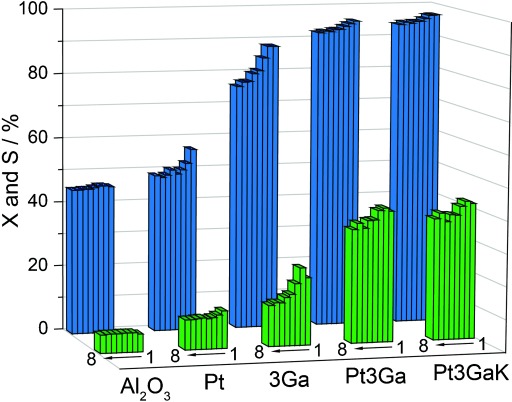
Conversion of propane (X, green) and selectivity (S, blue) for propene during PDH on the Al_2_O_3_ support, Pt, 3Ga, Pt3Ga, and Pt3GaK catalysts for each of the eight successive dehydrogenation cycles. The exact values of X and S for the first, second, and eighth cycle are summarized in Table 1. Evidently, both Pt and Ga are required to obtain a highly active selective and stable propane dehydrogenation catalyst.

**Table 1:** The conversion (X) and selectivity (S) obtained halfway through the first, second, and eighth cycle of the ten catalyst materials under investigation.[a]

Catalyst material (supported on Al_2_O_3_)	Code	First cycle	Second cycle	Eighth cycle	Coke dep[b]	Darkening[c]	D/G[d]			
		X [%]	S [%]	X [%]	S [%]	X [%]	S [%]	(wt %)	[%]	(−)
1000 ppm Pt, 1.5 wt % Ga, 0.25 wt % K	PtGaK	42.0	96.7	41.9	96.1	35.3	93.5	0.33	2.2	0.74
1000 ppm Pt, 3 wt % Ga, 0.25 wt % K	Pt3GaK	41.9	96.9	42.6	96.7	37.5	94.0	0.24	4.0	0.80
1000 ppm Pt, 1.5 wt % Ga	PtGa	42.0	96.7	42.6	96.8	32.3	92.8	0.55	10.8	0.84
1000 ppm Pt, 3 wt % Ga	Pt3Ga	40.3	95.4	41.3	94.6	35.0	91.9	0.52	20.6	0.73
1.5 wt % Ga, 0.25 wt % K	GaK	14.7	88.9	24.0	86.5	15.7	78.2	0.23	2.0	0.96
3 wt % Ga, 0.25 wt % K	3GaK	21.8	89.0	21.8	85.4	10.4	65.8	0.43	5.4	0.94
1.5 wt % Ga	Ga	15.8	81.3	20.4	84.8	15.3	77.4	0.34	33.5	0.98
3 wt % Ga	3Ga	20.7	88.0	23.8	88.0	12.6	75.7	0.33	12.5	1.00
1000 ppm Pt	Pt	11.5	56.4	10.3	52.1	9.2	48.5	0.44	1.1	0.64
bare alumina	Al_2_O_3_	5.5	45.5	5.9	45.8	5.6	44.7	0.46	2.3	0.78

[a] The catalysts show somewhat higher values for conversion at the start of a dehydrogenation cycle, which then slowly drop during the 15 min cycle. The wt % of coke deposited on the catalyst material as measured by TGA, the catalyst darkening as measured by operando UV/Vis and the D/G ratio as measured by operando Raman are also included.

[b] The coke deposited was calculated using the TGA curve with (α−*β*)/α^*^100 %, with α being w_cat_ at *T*=300 °C, and β being w_cat_ at *T*=650 °C.

[c] Darkening is defined as the light absorbed by the catalysts between 750 and 850 nm, relative to carbon nanofibers and the white catalyst.

[d] Ratio of the intensities of the D and G bands obtained from the operando Raman spectra.

From Table 1 and Figure 1, it can be concluded that the catalytic conversion varies strongly between the different catalyst materials. In case of the catalysts containing both Pt and Ga, this value is close to the equilibrium conversion (which is ca. 55 % at 620 °C and 1 atm pressure) for the first dehydrogenation cycle. When Pt is absent, the conversion is roughly halved, while the absence of Ga results in an even greater drop in conversion. At the same time, the selectivity is high for all catalysts containing both Pt and Ga, but decreases for the materials containing only Pt or Ga. This implies a synergy between Pt and Ga that results in a highly active and selective catalyst. Alkali metal dopants, such as K, are known to increase the propene selectivity and decrease coke deposition by poisoning the Brønsted acid sites present in the PDH catalysts.[9] Indeed, a slight increase is observed in propene selectivity after addition of K to the Pt and Ga containing catalysts. Finally, the bare support displays a very low activity and selectivity and is therefore regarded to be inactive. After the first PDH cycle, the conversion and selectivity of the catalysts does not drop, verifying that the catalysts are not deactivated. In fact, for the GaK, Ga, and 3Ga catalysts, the propane conversion even increases. This implies that these catalysts require an activation period, related to the exposure to oxygen at 750 °C. Indeed, by treating the catalyst with oxygen at 750 °C prior to the first propane dehydrogenation cycle, the conversion is increased from 14.7 to 20.5 % for the GaK catalyst. Treating the GaK catalyst at 620 °C under air prior to reaction has a lower impact and the conversion is only 18.5 % for the first propane dehydrogenation cycle. Apparently, the high temperature during the regeneration is required for the Ga to remain active in the PDH process.

For the eighth cycle, the values of propane conversion and propene selectivity have dropped for all catalysts. The deactivation is the least severe for those catalysts containing both Pt and Ga. It is known that Pt-based dehydrogenation catalysts deactivate due to sintering of the metal nanoparticles, an effect provoked by the harsh conditions of the dehydrogenation reaction.[10] Therefore, it is surprising that even though the dehydrogenation and oxidation are performed at relatively high temperatures in this study, no such deactivation is observed for the PtGa catalysts: the synergy between the Pt and Ga remains as these materials continue to outperform their analogues, which solely contain Ga.

In addition to the catalytic tests discussed in Figure 1 and Table 1, a long-term stability test consisting of approximately 150 cycles or 14 days of operation, was performed on the very active Pt3GaK catalyst. The catalytic performance of the catalyst during this experiment is shown in Figure 2. It was found that the PDH activity drops significantly during the first two days of testing, after which the catalyst performance remained stable during a twelve-day evaluation period, giving a propane conversion of 31.1 % and a selectivity for propene of 92.6 %, stressing the high stability of the catalyst material.

**Figure 2:**
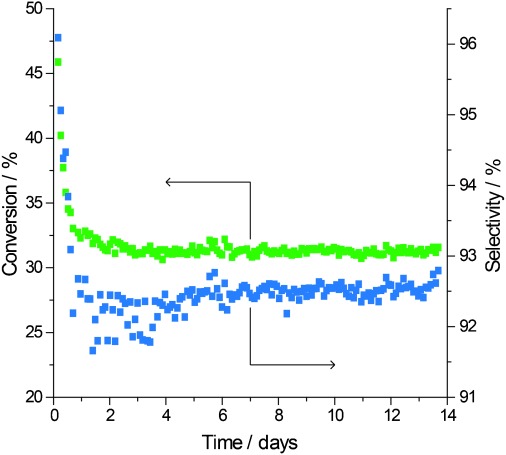
Long-term stability experiment with the Pt3GaK catalyst, which was cycled for ca. 150 times over a 14 day period. During the first two days both the conversion and selectivity drop, after which the catalyst performance remains stable for 12 days on stream.

Operando Raman and UV/Vis spectra have been collected during the catalytic dehydrogenation experiments and the results are summarized in Table 1. From the UV/Vis spectra (Figure S4), it is concluded that the absorption increases during the first minutes on stream for the PtGaK catalyst, after which the spectra do not change anymore. In the absence of K (PtGa), the darkening is a more gradual process that continues throughout the cycle. To compare the relative darkening of the different catalyst materials, an arbitrary darkening scale was designed, where 0 % darkening represented a pristine white catalyst, and 100 % darkening a completely coked catalyst, for which we used carbon nanofibers as the reference material. As Table 1 shows, the presence of K results in less darkening of the catalyst material, due to less coke being deposited. For each of the eight propane dehydrogenation cycles, a similar level of catalyst darkening is observed for all materials under study.

In Figure 3, the operando Raman spectra obtained at the end of the eighth propane dehydrogenation cycle are shown for the different investigated catalysts. Two Raman bands typical of coke are observed: the so-called D (disordered, at 1320 cm^−1^) and G band (graphitic coke, at 1590 cm^−1^). Specific information about the nature of the coke deposits formed on the catalyst surface can be obtained from the ratio of these two Raman bands.[11] Interestingly, the presence of Pt in the catalyst material has a significant effect on the D/G ratio. In Figure 3 the lighter colored spectra represent the coke formed on Pt-containing catalysts, whereas the darker colored spectra represent the coke on their non-Pt-containing counterparts. As the Raman spectra are normalized to the G band, it is clear that the D band is more intense for the catalyst materials that do not contain Pt. When looking at the D/G ratio shown in Table 1, the catalyst materials that contain Pt and Ga have a D/G ratio of around 0.75, while catalyst materials that do not have Pt in their composition have a D/G ratio of approximately 0.97. A possible explanation is that Pt further dehydrogenates the carbon deposits, leading to a higher graphitic portion in the coke. However, it should be noted that the coke deposited on the support also has a D/G ratio of 0.78. Similar values for the D/G ratios are obtained from the first dehydrogenation cycle, showing that the nature of the coke deposits does not change.

**Figure 3:**
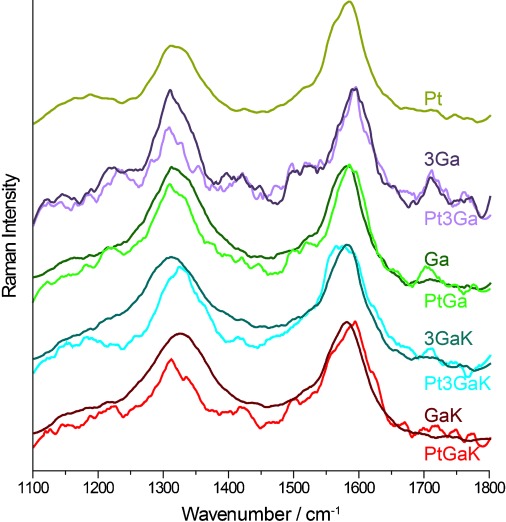
Operando Raman spectra obtained during the eighth propane dehydrogenation cycle for the different catalyst materials under investigation. The spectra are normalized with respect to the G band at 1600 cm^−1^. The lighter colored Raman spectra are for the Pt-containing catalysts; the darker spectra of their non-Pt-containing counterparts.

After the eighth cycle, the catalyst materials were not regenerated, but instead collected from the reactor and analyzed by thermogravimetric analysis (TGA) combined with on-line mass spectrometry (MS) to investigate the coke deposits formed. The change of weight per temperature interval as a function of temperature is shown in Figure S5. In addition, during the combustion a distinct CO_2_ profile is observed for each catalyst by on-line MS analysis, which is shown in Figure S6. These profiles accurately describe the temperature where coke is combusted, which is between 300–650 °C; the weight loss corresponding with this temperature interval is included in Table 1. The amount of CO_2_ detected is very small for the catalyst containing only Pt, for the bare support, and for the catalysts containing K. Less coke is therefore deposited on these catalysts, in agreement with what was observed with operando UV/Vis spectroscopy. On the other hand, the absence of K and the presence of Ga results in significant amounts of coke on the catalyst surface. As Brønsted acidity is associated with the deposition of coke, the presence of GaO_*x*_ may introduce acidity to the catalyst surface, resulting in catalyst coking. When K is present on the catalyst, these sites are poisoned, inhibiting the formation of coke. Note that this implies that almost no acidity is present on the bare support to start with.

The specific nature of the gallium species present on the catalyst was investigated by collecting ^71^Ga MAS NMR (magic-angle spinning NMR) and XPS (X-ray photoelectron spectroscopy) spectra of the fresh Pt3Ga and 3Ga catalysts (Figure 4, Figure S7, and Table 2). In the NMR spectra, two peaks with chemical shifts of 151 and 15 ppm are observed (relative to the signal of Ga(NO_3_)_3_), corresponding to tetrahedrally (IV) and octahedrally (VI) coordinated Ga^3+^, respectively.[12] The spectra show a strong resemblance to ^71^Ga MAS NMR spectra of a ternary oxide composed of Ga, Al, and O, as reported by Chen et al.[13] Such a mixed oxide is likely formed during the high temperatures (750 °C) of the calcination step after impregnation. Chen et al. proposed that a spinel structure is formed, in which Ga^3+^ is preferentially located in a tetrahedral coordination. Such a tetrahedral preference of Ga^3+^ has been reported for several mixed oxides containing Ga and is explained by a covalent contribution to the metal oxygen bond caused by the so-called d-block contraction. As the d-orbital becomes completely filled, it ineffectively shields the nuclear charge, resulting in a higher polarization power.

**Figure 4:**
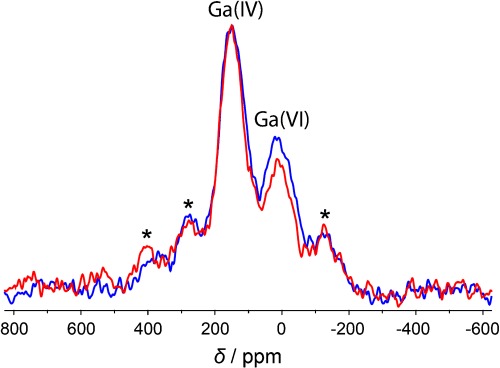
^71^Ga MAS NMR spectra of the fresh Pt3Ga (red) and 3Ga (blue) catalyst materials. Asterisks denote the spinning sidebands.

**Table 2:** Chemical composition of the surface of the 3Ga and Pt3Ga catalysts as measured by XPS.[a]

Catalyst	Al (wt %)	O (wt %)	Ga (wt %)	Pt (wt %)
3Ga	54.60	41.29	4.11	0
Pt3Ga	54.20	39.79	4.67	1.33

[a] The concentrations of the elements present at the surface are expressed in wt %, which allows for a more clear comparison between the surface composition and the catalyst composition as a whole.

XPS measurements on the fresh samples of the Pt3Ga and 3Ga catalysts showed that only Ga^3+^ is present and that the concentration of Ga on the surface is only marginally higher as compared to the catalyst as a whole (Table 2; 4.11 wt % observed versus 3 wt %). This indicates that a significant amount of Ga is incorporated in the bulk of the support, which confirms the observation made by NMR that a mixed Al_2_O_3_–Ga_2_O_3_ oxide is formed. On the contrary, the apparent concentration of Pt is high on the surface (1.3 wt % observed versus 1000 ppm), suggesting that the Pt is well dispersed on the surface. Furthermore, the presence of Pt appears to affect the distribution of Ga on the catalyst material. First of all, the concentration of Ga on the catalyst surface is higher for the Pt3Ga catalyst compared to the 3Ga catalyst (4.67 wt % versus 4.11 wt %). Secondly, from the ^71^Ga MAS NMR spectra of these two catalysts (Figure 4), it is observed that a larger amount of tetrahedrally coordinated Ga^3+^ is present in Pt3Ga compared to the 3Ga catalyst. This suggests that the presence of Pt results in a more tetrahedral Ga^3+^ species on the catalyst surface. The respective XPS spectra are discussed in more detail in the Supporting Information (Figure S7).

Because metallic Ga is a liquid and Ga_2_O a volatile compound, it is important to consider the reducibility of Ga^3+^, especially because the dehydrogenation reaction is performed at high temperatures in a reducing atmosphere. Alternatively, a Pt–Ga alloy may be formed by hydrogen spillover from the Pt, whereby Ga^3+^ is reduced to Ga^0^, which then forms the alloy.[7,14] TPR and quasi-in situ XPS was employed to investigate the reducibility of the 3Ga and Pt3Ga catalysts. The temperature-programmed reduction (TPR) experiment showed that no hydrogen was being consumed while the catalyst was heated up to 700 °C under a constant hydrogen flow (Figure S8). For the XPS experiment, the catalyst was reduced in a reactor, after which it was loaded into the XPS apparatus without being exposed to air. Again, no reduced Ga species were detected (Figure S9). Apparently, the mixed Ga–Al oxide is too stable to be reduced at these conditions, even in the presence of Pt.

CO chemisorption was used to study the effect of elevated temperatures on the Pt dispersion in reducing or oxidizing environments. The PtGaK catalyst was heated stepwise under either H_2_ or air at 350, 550, and 650 °C, without removing it from the chemisorption unit (Figure S10). The Pt dispersion does not change, with an exception when the catalyst is treated at 650 °C under air; in this case the dispersion quickly collapses. During the regeneration step, the catalyst is treated at 750 °C under air, which would have similar effects on the Pt dispersion. Therefore, the Pt surface area does not correlate with the activity of the catalyst, meaning Pt is not the species mainly responsible for the PDH activity of the catalyst.

Combining the trends observed with ^71^Ga NMR and XPS, the presence of Pt results in a higher concentration of surface tetrahedral Ga^3+^ species. Nevertheless, such a relatively small increase in active sites cannot account for the high activity observed for the Pt3Ga catalyst, compared to 3Ga. Since the only Pt-containing catalyst is almost inactive in the dehydrogenation reaction and the Pt dispersion drops severely after treatment under air at elevated temperatures, it is assumed that coordinately unsaturated Ga^3+^ species are responsible for the C=H bond activation.[15] The proposed reaction mechanisms for the dehydrogenation on Ga_2_O_3_ catalysts are discussed in a review by Copéret, in which he states that the dissociative adsorption of propane results in the formation of a surface hydroxy group and either a Ga alkyl or a Ga alkoxy species.[16] After the elimination of the β-hydrogen through the formation of hydride or a second hydroxy group, propene desorbs. However, as Pidko et al. have pointed out, the subsequent regeneration of the active sites through the formation of hydrogen is problematic, because the reduction of Ga^3+^ to Ga^+^ and H_2_O is energetically more favored.[17] For our catalyst material, the mixed Al–Ga oxide is too stable to be reduced, meaning that the GaH/GaOH species need to be regenerated. We postulate that the Pt assists in the recombination of the hydrogen atoms on the catalyst, making the active sites available for the following dehydrogenation cycle.

Finally, the Pt3GaK catalyst was compared with a wide range of other catalyst materials reported in literature, as well as a commercial CrO_*x*_ catalyst (Figure S11). When comparing the propylene yield with the weight hourly space velocity, the Pt3GaK catalyst displays a superior activity, further highlighting the excellent catalytic performance of the material.

In summary, different Pt-Ga-K-containing catalyst materials have been examined for the selective dehydrogenation of propane into propene and it was found that the combination of 1000 ppm Pt and 1.5–3 wt % Ga results in a highly active and selective catalyst. The catalyst is highly resistant to coking and remains active for prolonged reaction times. A combination of structural, morphological, and surface characterization reveals a complex catalyst material with a synergistic and bifunctional character originating from the supported Ga and Pt moieties, with Ga performing the actual dehydrogenation reaction and Pt being a unique promoting element.

## Experimental Section

The catalyst materials under investigation have been prepared by the incipient wetness impregnation method using Pt(NH_3_)_4_(NO_3_)_2_ (99.995 %), Ga(NO_3_)_3_ (99.9 %), and KNO_3_ (>99 %) as metal precursors and alumina as the support material. After impregnation, the catalyst is calcined at 750 °C under air. The catalyst materials have been characterized by a variety of techniques. Bright-field TEM analysis has been performed on a Tecnai 20 apparatus equipped with a field emission gun at 200 keV. XRD diffractograms were collected with a Bruker D2 Phaser, equipped with a Co (Kα) anode. For the N_2_-physisorption experiments a TriStar 3000 V6.08 A has been used at −196 °C after drying the samples overnight. Catalytic tests have been performed on a reactor setup, which allows for combined operando UV/Vis, Raman, and on-line GC analysis.[18] A cylindrical quartz tube equipped with optical grade windows was loaded with 0.150 g of catalyst material. The reaction was run at 620 °C with a flow of 9 mL min^−1^ of propane for 15 min, followed by a regeneration step at 750 °C with a flow of 6 % O_2_ in He for 30 min. During these reaction steps, operando UV/Vis and Raman spectra were collected by an Avantes 2048 UV/Vis spectrometer (50 accumulations and 70 ms exposure time) and a Kaiser Optical Systems Raman spectrometer (7 accumulations and 5 s exposure time), respectively. The reaction stream was analyzed by an on-line GC, which was equipped with a flame ionization detector (FID; Porabond-Q column) and a thermal conductivity detector (TCD; Carboxan column). Coked catalyst samples obtained after eight dehydrogenation cycles were examined on a PerkinElmer Pyris 1 TGA instrument. Between 10 and 25 mg of catalyst material was dried at 150 °C under an Ar flow and then heated under a flow of O_2_ from 30 to 900 °C at a ramp of 10 °C min^−1^. The gas stream exiting the TGA apparatus was analyzed by an Omnistar mass spectrometer from Pfeiffer Vacuum. The ^71^Ga MAS NMR experiments were performed in a 9.4 T Bruker Avance III NMR system using an MAS rate of 16 kHz. To minimize baseline distortions, a windowless spin-echo pulse sequence was implemented, with an echo delay of 2 μs and a dead time of 5 μs.[19] The radio frequency field strength was set to 83 kHz and experiments conducted lasted for 12 days. XPS experiments were performed on a Thermo Scientific K-Alpha apparatus, equipped with an Al Kα (1486.6 eV) X-ray anode. The catalysts were deposited on a carbon sticky tape in order to prevent charging. For analyzing the XPS spectra, the CasaXPS program is used. For the quasi-in situ experiment, the Pt3GaK catalyst was reduced for 1 h under a H_2_ flow at 620 °C, after which the sample was transferred to a Kratos AXIS Ultra spectrometer, equipped with a monochromatic X-ray source and a delay-line detector (DLD). Spectra were obtained using the aluminum anode (Al K*α*=1486.6 eV) operating at 150 W and a background pressure of 2×10^−9^ bar. TPR experiments were performed on a Micromeritics Autochem II flow system, equipped with a TCD detector. 0.25 g of catalyst is placed in a quartz tube, after which the sample is dried prior to being heated to 700 °C under a flow of 5 % H_2_ in He. A Micromeritics ASAP 2020 Accelerated Surface Area and Porosimetry System was used to analyze the Pt metal dispersion using CO adsorption. 1.8 g of sample supported on a bed of quartz wool was loaded in a quartz sample tube and inserted in the instrument. The sample was pretreated prior to the chemisorption experiment by flushing nitrogen for 10 min at 35 °C, oxidizing the sample in a 10 %-oxygen-in-helium atmosphere at varying temperatures (10 °C min^−1^ ramp) for 240 min, a reducing treatment in hydrogen at varying temperatures (10 °C min^−1^) for 240 min, and evacuating (5 μm Hg) for 60 min at reaction temperature. The sample was analyzed with carbon monoxide at 35 °C with 15 pressure points from 25 mm Hg to 650 mm Hg. After completing the first isotherm, the sample was evacuated (10 μm Hg) for one hour at 35 °C, after which a second isotherm was collected at the same conditions. The metal dispersion was calculated based on the difference of these isotherms extrapolated to 0 mm Hg, assuming a unitary ratio of carbon monoxide to surface Pt.
