# Supplementary material for: Platinum-Promoted Ga/Al2O3 as Highly Active, Selective, and Stable Catalyst for the Dehydrogenation of Propane
Source: Angew Chem Int Ed Engl. 2014 Jul 2;53(35):9251–6. doi: 10.1002/anie.201404460 (PMC4499260; doi:10.1002/anie.201404460)
Supplement: Supplementary file 1 [file anie0053-9251-sd1.pdf]

Angewandte Chemie
Eine Zeitschrift der Gesellschaft Deutscher Chemiker

Supporting Information

© Wiley-VCH 2014

69451 Weinheim, Germany

Platinum-Promoted Ga/Al_2O_3 as Highly Active, Selective, and Stable Catalyst for the Dehydrogenation of Propane**

Jesper J. H. B. Sattler, Ines D. Gonzalez-Jimenez, Lin Luo, Brien A. Stears, Andrzej Malek, David G. Barton, Beata A. Kilos, Mark P. Kaminsky, Tiny W. G. M. Verhoeven, Eline J. Koers, Marc Baldus, and Bert M. Weckhuysen**

anie_201404460_sm_miscellaneous_information.pdf

Supplementary Information

1. Standard Characterization

After preparation, the catalyst materials were analysed by XRD, TEM and N_2-physisorption. Figure S1 shows the obtained X-ray diffractograms of the bare support and the PtGaK and Pt3GaK catalysts. It can be concluded only diffraction lines corresponding with the alumina support are observed. Ga and K are likely to be too well dispersed over the catalyst material to show up as crystalline phases, while the concentration of Pt is too low to be able to detect Pt nanoparticles with the XRD technique.

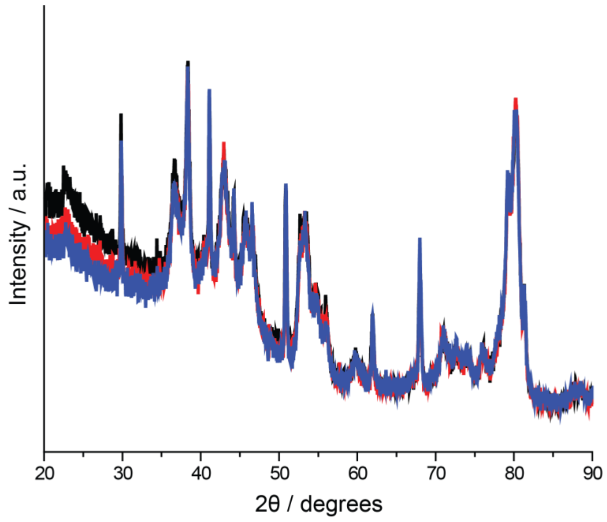

Figure S1. X-ray diffraction (XRD) patterns of the bare support (black) and the PtGaK (red) and Pt3GaK catalysts (blue).

Figure S2 shows a Transmission electron microscope (TEM) image of a PtGaK catalyst particle of about 200 nm in diameter. By analysis of multiple particles, no Pt or Ga_2O_3 crystallites were detected, indicating a high dispersion and small particle size. This was confirmed by energy dispersive X-ray linescans (EDX), which revealed both elements were dispersed homogeneously over the support.

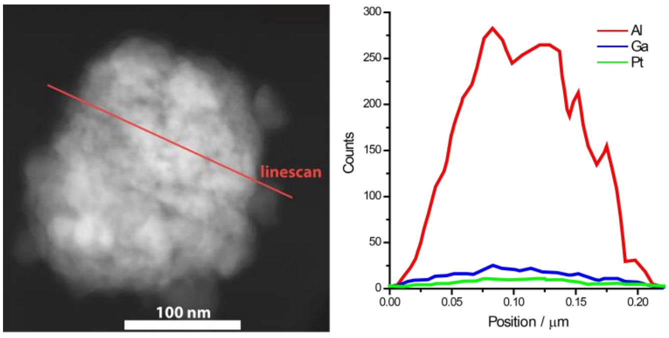

Figure S2. TEM image of a PtGaK catalyst particle (left) along with an EDX linescan, corresponding with the red line shown (right).

Table S1. Pore volume and surface area related to the Ga loading as measured by N_2-physisorption.

Catalyst	Pore Volume (ml/g)	BET surface area (m^2/g)
Alumina	0.24	73.8
PtGaK	0.23	71.2
Pt3GaK	0.24	74.8

In Table S1 the surface area and pore volume obtained by N_2-physisorption are tabulated for the bare support as well as the PtGaK and Pt3GaK catalysts. It can be concluded that impregnation of the support oxide did not significantly alter the surface area nor pore volume of the catalyst material.

2. GC analysis

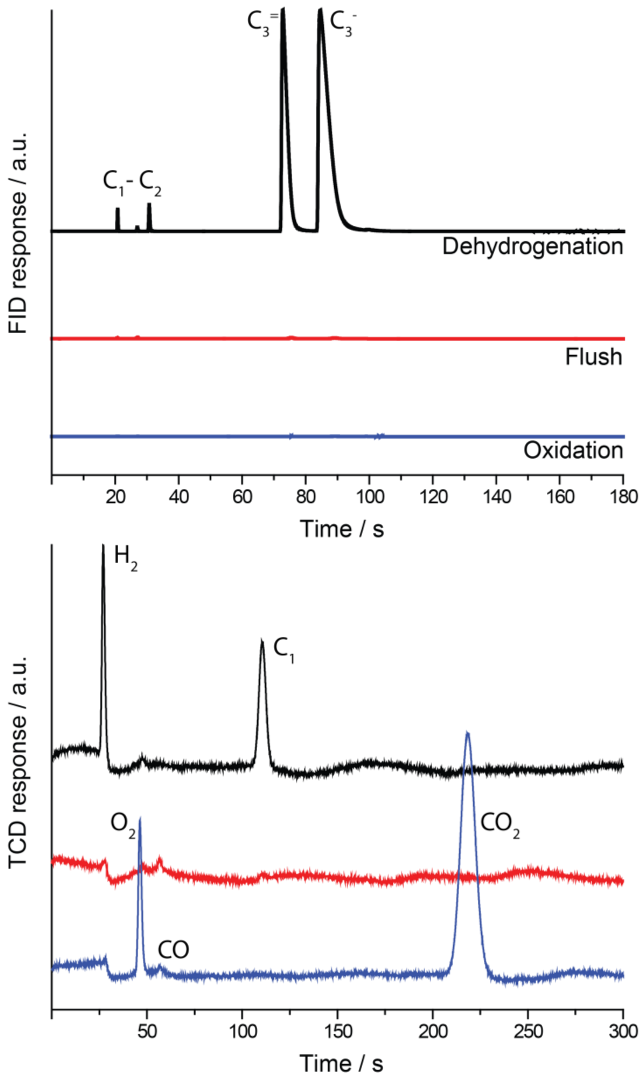

Figure S3. Gas chromatograms collected during the dehydrogenation (black), flush (red) and oxidation steps (blue) for both the flame ionization detector (FID, top) and thermal conductivity detector (TCD, bottom).

The conversion and selectivity of the catalyst materials were determined by on-line GC analysis. In Figure S3, typical chromatograms obtained during the dehydrogenation, flush and oxidation steps on the Pt3Ga catalysts are shown. At the top, the combustible compounds detected with a flame ionization detector (FID) are shown. On the bottom, chromatograms obtained with a thermal conductivity detector are shown, which is used to detect the other gasses in the feed. It can be observed that during the dehydrogenation step other than H_2, propane and propene, only cracking or hydrogenolysis products are detected. During the oxidation step O_2 and CO_2, as well as trace amounts of CO are measured.

3.Operando Raman and UV-Vis Spectroscopy

During the catalytic performance measurements, operando Raman and UV-Vis spectra have been collected in a continuous fashion. As an example, in Figure S4 operando UV-Vis spectra obtained during the eighth dehydrogenation cycle on the PtGa and PtGaK catalysts are presented. For the PtGaK catalyst, the absorbance only slightly increases during the dehydrogenation step, suggesting very little coke deposition. In addition, this laydown of coke occurs during the first few minutes of the reaction, after which the UV-Vis spectra remain the same. As this darkening is attributed to the laydown of carbon deposits, the presence of potassium appears to inhibit coke formation. Similar spectra were obtained on the other catalysts containing potassium. To compare the relative darkening of the different catalyst materials, an arbitrary darkening scale was designed, where 0% darkening represented the pristine white reference (Absorbance of 0), and 100% darkening (Absorbance of 0.8) a completely coked catalyst, for which we used carbon nanofibers as the reference material. Next the absorption at the start and end of the dehydrogenation cycle was determined by averaging all the data points between 750 and 850 nm, and the obtained value for absorbance was expressed as a percentage. Catalyst formulations in which potassium was excluded, showed more darkening that also increased gradually over the course of the dehydrogenation cycle, as is shown in the bottom of Figure S4. These catalysts exhibit a dark colour, unlike the catalysts that contain potassium, who remain white. Simultaneously, operando Raman spectra are collected during the dehydrogenation step. In Figure 3 the spectra obtained during the eighth dehydrogenation cycle are shown. In the Raman spectra, four separate bands can be distinguished: two bands corresponding with the quartz reactor window (1040 and 1166 cm^{-1}) and two bands corresponding with carbon deposits. The first band, the G or graphite band corresponds with a perfect graphitic lattice. The second band, the so-called D-band or disordered coke band, corresponds with defects and edges in the graphite lattice. The ratio between the intensities of these two bands reveals the level of graphitisation of the coke on the catalyst. Lorentzian line shapes are used to fit the bands in order to determine the intensity accurately. As is shown in Table 1, the presence of Pt has a significant effect on the type of coke deposited on the catalyst. A D/G ratio of approximately 0.75 is measured, compared to on an average D/G ratio of 0.97 for the catalyst materials without Pt present, meaning more graphitic coke is deposited in the presence of Pt. The noise in the spectra is explained by a strong fluorescent background, and the relatively low concentration of the coke.

Summarizing, by operando UV-Vis spectroscopy we can follow catalyst darkening over time, which provides indirect information of the rate of coke deposition and therefore represents a rough measure of the amount of coke deposited. In the presence of potassium, a small amount of coke is deposited in the first minutes of the reaction, after which further coke deposition is inhibited. On the contrary, in the absence of K coke deposition is a gradual process that continues over the course of the PDH cycle. Operando Raman spectroscopy provides information on the chemistry of the coke deposits. In our experiments it was shown that in the presence of Pt, more graphitic coke deposits are formed.

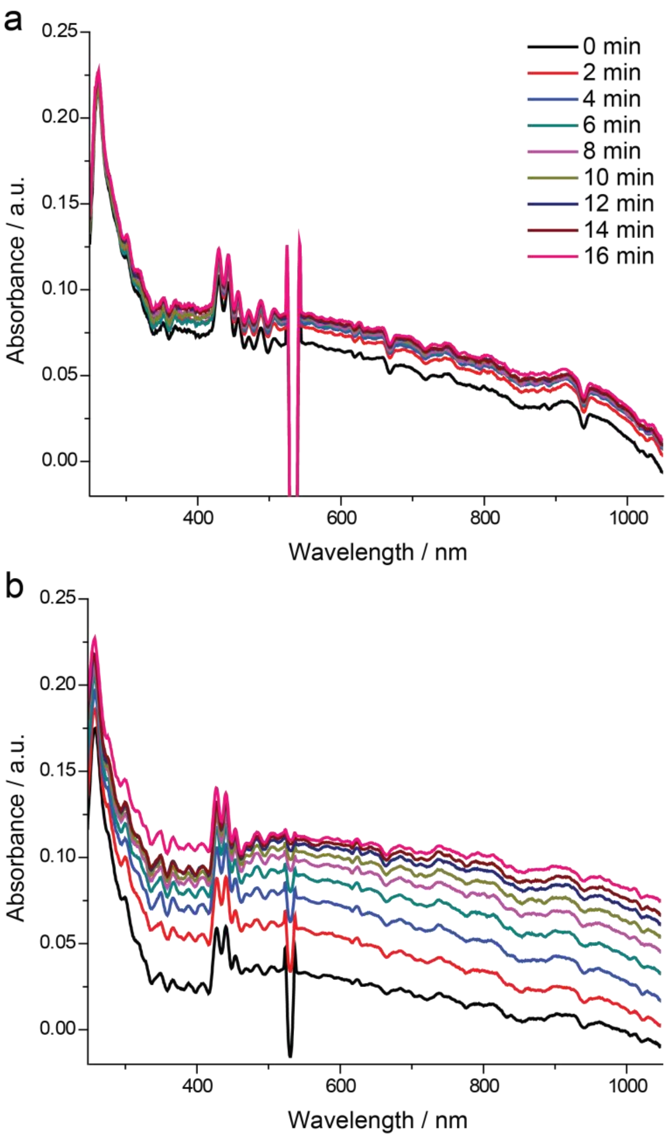

Figure S4. Operando UV-Vis spectra obtained during the 8th dehydrogenation cycle on the PtGaK (a) and PtGa (b) catalysts. The feature at 532nm in the spectrum is caused by diffusely scattered laser light from the Raman light source

4. Thermogravimetrical Analysis

In Figure S5 the differentiated weight loss as a function of time is shown for the different deactivated catalysts. All catalysts, including the bare support, lose mass continuously during the TGA experiment. Between 350 and 600 ^{0}C peak in the mass loss is observed for the 1.5Ga, Pt1.5Ga, 3Ga and Pt3Ga catalysts, which is attributed to the combustion of the coke deposits. A strong increase in the concentration of CO_2 is observed by the MS analysis at the same temperatures, as is shown in Figure S6. In the bottom of Figure S5 and S6, only a very small signal corresponding with the combustion of coke is observed for the K containing catalysts. For the different catalysts measured, the CO_2 signal is centred at 475^{0}C, with the exception of the catalyst containing only Pt, which is positioned around 590^{0}C. The temperature where combustion occurs is related to the chemical nature of the coke and the relative position of species in respect to the catalytic centres: on one hand, if the coke deposits are more graphitic, they require higher temperatures to be combusted, while on the other hand proximity to catalytically active centres can catalyse combustion [20]. From operando Raman spectroscopy it was determined that the presence of Pt results in more graphitic species formed on the catalyst. However, this cannot account for the higher combustion temperature of the coke formed on the 1000 ppm Pt catalyst, as for example no clear difference is observed between the TGA data of the Pt1.5Ga and 1.5Ga catalysts. Possibly, the presence of Ga is important to catalyse the combustion of coke. On the other hand Pt may be completely covered by coke and can therefore not assist in the combustion of the carbon species.

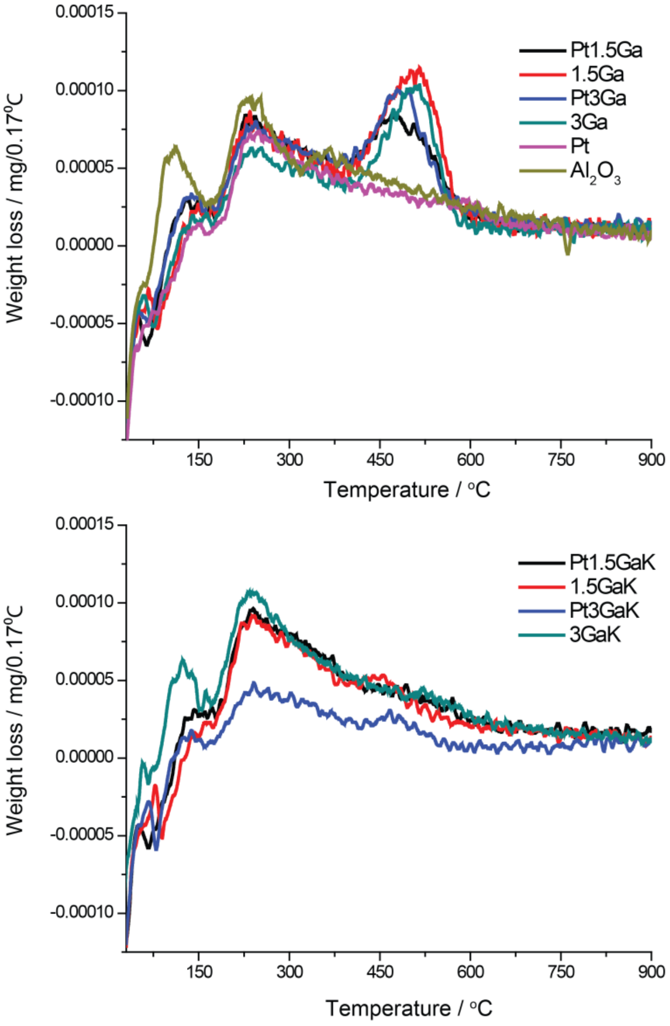

Figure S5. Weight loss of the catalyst per 0.17^{0}C interval for the catalyst materials under study. For PtGa, Ga, Pt3Ga and 3Ga a strong additional loss of weight is measured around 500^{0}C, which is much weaker for the related catalysts containing K

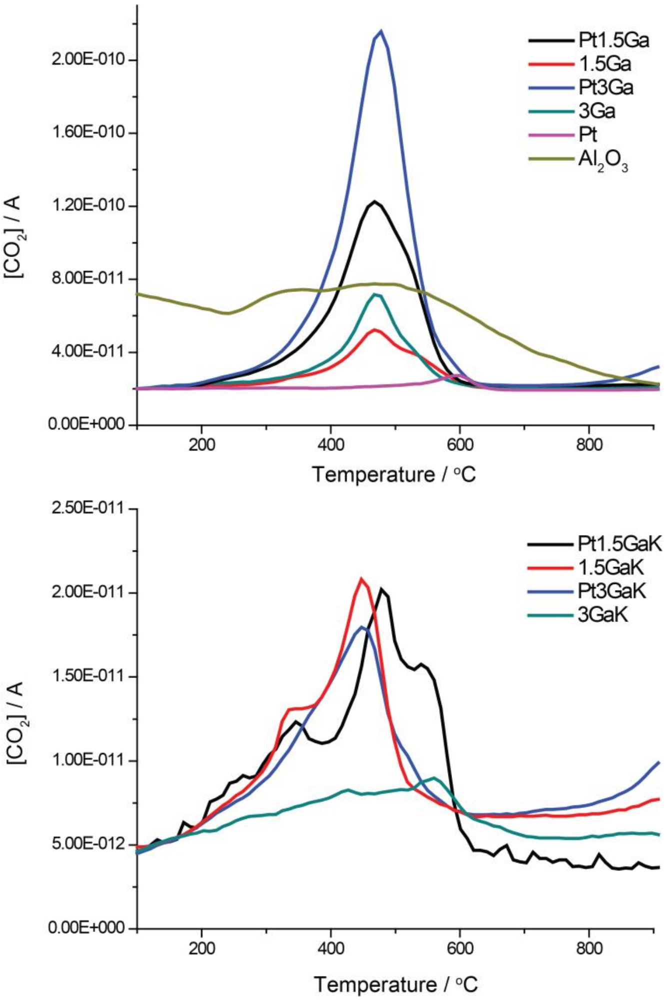

Figure S6. CO_2 concentration profiles as a function of reaction temperature as measured by MS analysis during the TGA combustion step. The samples discussed here are deactivated catalyst materials obtained after the 8th dehydrogenation cycle of the experiments described in Table 1. In the top Figure, catalysts containing K, and at the bottom catalysts without K are shown. Note that the MS has not been calibrated between every experiment, which is why a quantitative comparison between the different experiments is not possible.

5. XPS analysis

In Figure S7, XPS spectra of the Al 2p, Ga 3d and Ga 2p core levels are shown of the Pt3Ga and 3Ga catalysts. The fitting procedure was as the following: First of all, the bare Al_2O_3 support was measured (not shown) and the parameters obtained were used to fit the oxygen and aluminium species of the 3Ga and Pt3Ga catalysts. As the Al 2p peak shows an overlap with the Pt 4f peak, the known ratio between the Al 2s and Al 2p is used to fit the Al signal, and the remainder is used to fit the Pt 4f signal. Again, the known ratio between the Pt 4f 5/2 and Pt 4f 7/2 is used for fitting. The Pt signal obtained corresponds with metallic Pt (70.8 and 74.0 eV). However, the presence of oxidic Pt cannot be excluded (PtO 4f 7/2 = 72.9 eV; PtO_2 4f 7/2 = 74.5 eV), as the incident X-rays may reduce the Pt species present on the catalyst. Both the Ga 3d (20.6 eV) and Ga 2p signals (1119.0eV) correspond with Ga(III): no evidence of Ga(I) (19.1 eV; (-)) or metallic Ga (18.5 eV; 1115.6 eV) is observed [21]. For fitting the Ga 3d signal, a similar procedure as for the Al 2p edge is used. In addition, no reduced Ga species are observed after treating the 3Ga and Pt3Ga catalysts for 1 h in a hydrogen atmosphere at 620°C (Figure S9).

6. TPR analysis

To further examine the reducibility of the 3Ga and Pt3Ga catalysts, TPR experiments were performed, shown in Figure S8. During the experiment no consumption of hydrogen is observed, meaning no Ga species are being reduced, or the concentration is very low. Also no clear evidence of the reduction of Pt is observed, which may be related to the low concentration.

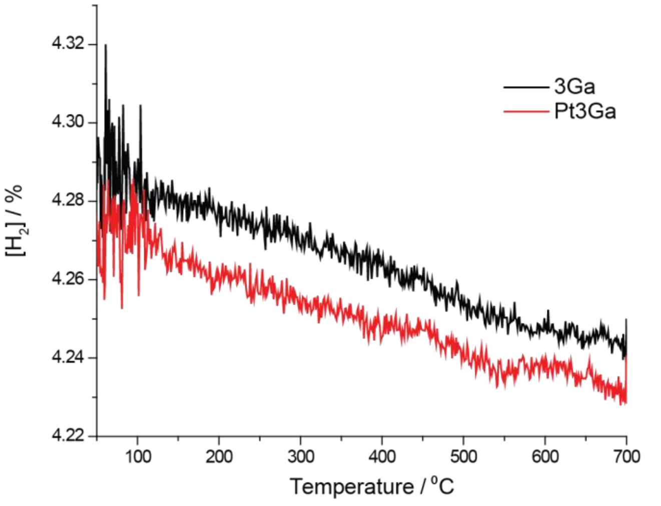

Figure S8. H_2 concentration versus temperature during the temperature programmed reduction experiment. The lack of features in the graph indicates no reduction is taking place.

7. CO Chemisorption

A program consisting of 7 separate steps was designed to study the Pt dispersion of the PtGaK catalyst as a function of temperature and gas atmosphere. After each step, the sample is cooled to ambient temperatures and the dispersion measured by CO chemisorption. In addition, each time the catalyst is exposed to air, the material is reduced by H_2 at 350°C, to reduce any PtO_x species present. During step 2, the catalyst is treated at 550°C under air, which results in a small increase in Pt dispersion. However when the catalyst material is treated at 650°C in air (step 3 and step 5), the Pt dispersion drops dramatically, which is in accordance to what has been reported in literature for Pt/Al_2O_3 [22]. On the contrary, the Pt dispersion stays stable when the catalyst is treated under hydrogen at the same temperature (step 4 and 6). Finally, by treating the catalyst again at 550°C (step 7), the Pt dispersion remains low, meaning the sintering of the Pt is irreversible.

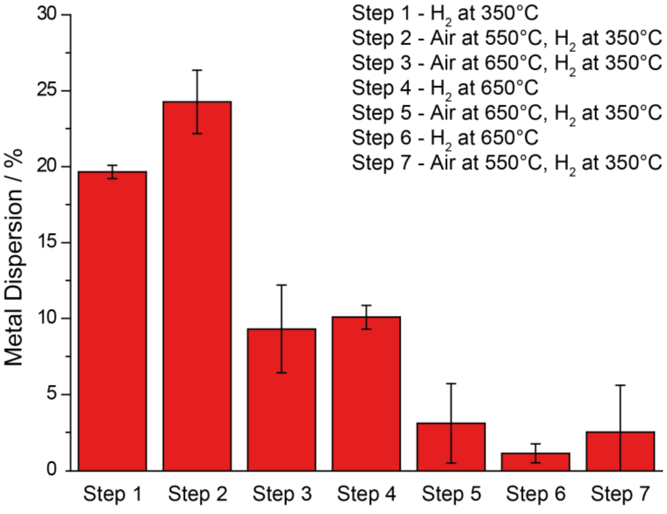

Figure S10. Pt dispersion of the PtGaK catalyst after the material is thermally treated under air and H_2 at elevated temperatures. The Pt dispersion drops after step 3 and 5, where the material is treated under air at 650°C. Treating the material under air at 550°C, or under hydrogen at 650°C has no effect on the Pt dispersion.

8. Comparison Pt3GaK to CrO_x/Al_2O_3

To prove the outstanding performance of this catalyst system, the Pt3GaK catalyst was compared to a list of propane dehydrogenation catalysts published in literature (note that the experimental conditions are not necessarily identical), and a commercial-like CrO_x catalyst, described in an earlier publication by Bennici et al. (reaction performed at identical reaction conditions). To highlight the excellent activity of the catalyst material, the initial yield at the start of the experiment is plotted versus the weight hourly space velocity, and is shown in Figure S11. A wide variety of catalyst materials from the literature was used for this comparison, including ones based on GaO_x, Pt, CrO_x, InO_x and carbon. It is clear the Pt3GaK catalyst is one of the best catalysts reported in terms of offering a high propylene yield at a relatively high weight hourly space velocities.

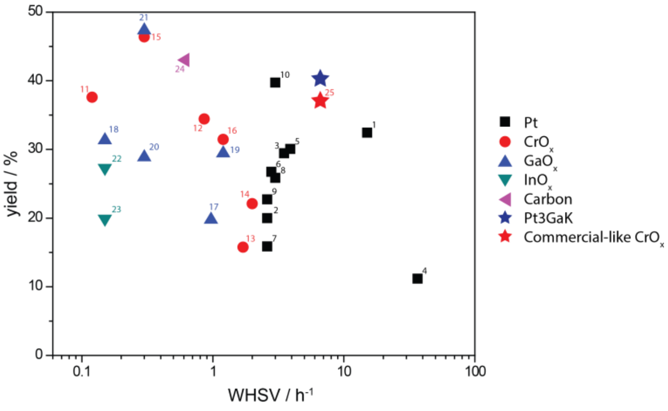

Figure S11. WHSV plotted against the yield (at t=0) for 24 catalyst materials reported in literature, a commercial-like CrO_x catalyst and the Pt3GaK catalyst material. Numbers adjacent to each data point correspond with the source where the activity data was obtained from. In all cases, the most active material from that publication was plotted in the Figure:

[1] T. Waku, J.A. Biscardi, E. Iglesia, *Chem. Commun.* **2003**, *9*, 1764-1765.

[2] P. Sun, G. Siddiqi, W.C. Vining, M. Chi, A. T. Bell, *J. Catal.* **2011**, *282*, 165-174.

[3] O. A. Barias, A. Holmen, E. A. Blekkan, *J. Catal.* **1996**, *158*, 1-12.

[4] J. Salmones, J. A. Wang, J. A. Galicia, G. Aguilar-Rios, *J. Mol. Catal. A-Chem.* **2002**, *184*, 203-213.

[5] Y. Zhou, M. . Davis, U.S. Patent 5214227. 5214227, 1993.

[6] S. A. I. Barri, R. Tahir, U.S. Patent 5126502. 5126502, 1992.

[7] G. Siddiqi, P. Sun, V. Galvita, A. T. Bell, *J. Catal.* **2010**, *274*, 200-206.

[8] Y. Duan, Y. Zhou, Y. Zhang, X. Sheng, M. Xue, M. *Catal. Lett.* **2011**, *141*, 120-127.

[9] P. L. De Cola, R. Gläser, J. Weitkamp, J. *Appl. Catal. A-Gen.* **2006**, *306*, 85-97.

[10] Y. Zhang, Y. Zhou, L. Huang, M. Xue, S. Zhang, *Ind. Eng. Chem. Res.* **2011**, *50*, 7896-7902.

[11] J. J. H. B. Sattler, I. D. González-Jiménez, A. M. Mens, M. Arias, T. Visser, B. M. Weckhuysen, *Chem. Commun.* **2013**, *49*, 1518-1520.

[12] K. Fujdala, T. D. Tilly, *J. Catal.* **2003**, *218*, 123-134.

[13] F. J. Pérez-Reina, E. Rodríguez-Castellón, A. Jiménez-López, *Langmuir* **1999**, *15*, 8421-8428.

[14] M. Alcántara-Rodríguez, E. Rodríguez-Castellón, A. Jiménez-López, *Langmuir* **1999**, *15*, 1115-1120.

[15] X. Zhang, Y. Yue, Z. Gao, *Catal. Lett.* **2002**, *83*, 19-25.

[16] P. Michorczyk, P. Pietrzyk, J. Ogonowski, J. *Micropor. Mesopor. Mat.* **2012**, *161*, 56-66.

[17] M. Saito, S. Watanabe, I. Takahara, M. Inaba, K. Murata, *Catal. Lett.* **2003**, *89*, 213-217.

[18] B. Zheng, W. Hua, Y. Yue, Z. Gao, *J. Catal.* **2005**, *232*, 143-151.

[19] P. Michorczyk, J. Ogonowski, *Appl. Catal. A-Gen.* **2003**, *251*, 425-433.

[20] B. Xu, B. Zheng, W. Hua, Y. Yue, Z. Gao, *J. Catal.* **2006**, *239*, 470-477.

[21] M. Chen, J. Xu, F. Su, Y. Liu, Y. Cao, H. He, K. Fan, *J. Catal.* **2008**, *256*, 293-300.

[22] M. Chen, J. Xu, Y. Liu, Y. Cao, H. He, J. Zhuang, *Appl. Catal. A-Gen.* **2010**, *377*, 35-41.

[23] M. Chen, J. Wu, Y. Liu, Y. Cao, L. Guo, H. He, K. Fan, *Appl. Catal. A-Gen.* **2011**, *407*, 20-28.

[24] L. Liu, Q. Deng, B. Agula, X. Zhao, T. Ren, Z. Yuan, *Chem. Commun.* **2011**, *47*, 8334-8336.

[25] S. M. Bennici, B. M. Vogelaar, T. A. Nijhuis, B. M. Weckhuysen, *Angew. Chem. Int. Edit.* **2007**, *46*, 5412-5416.

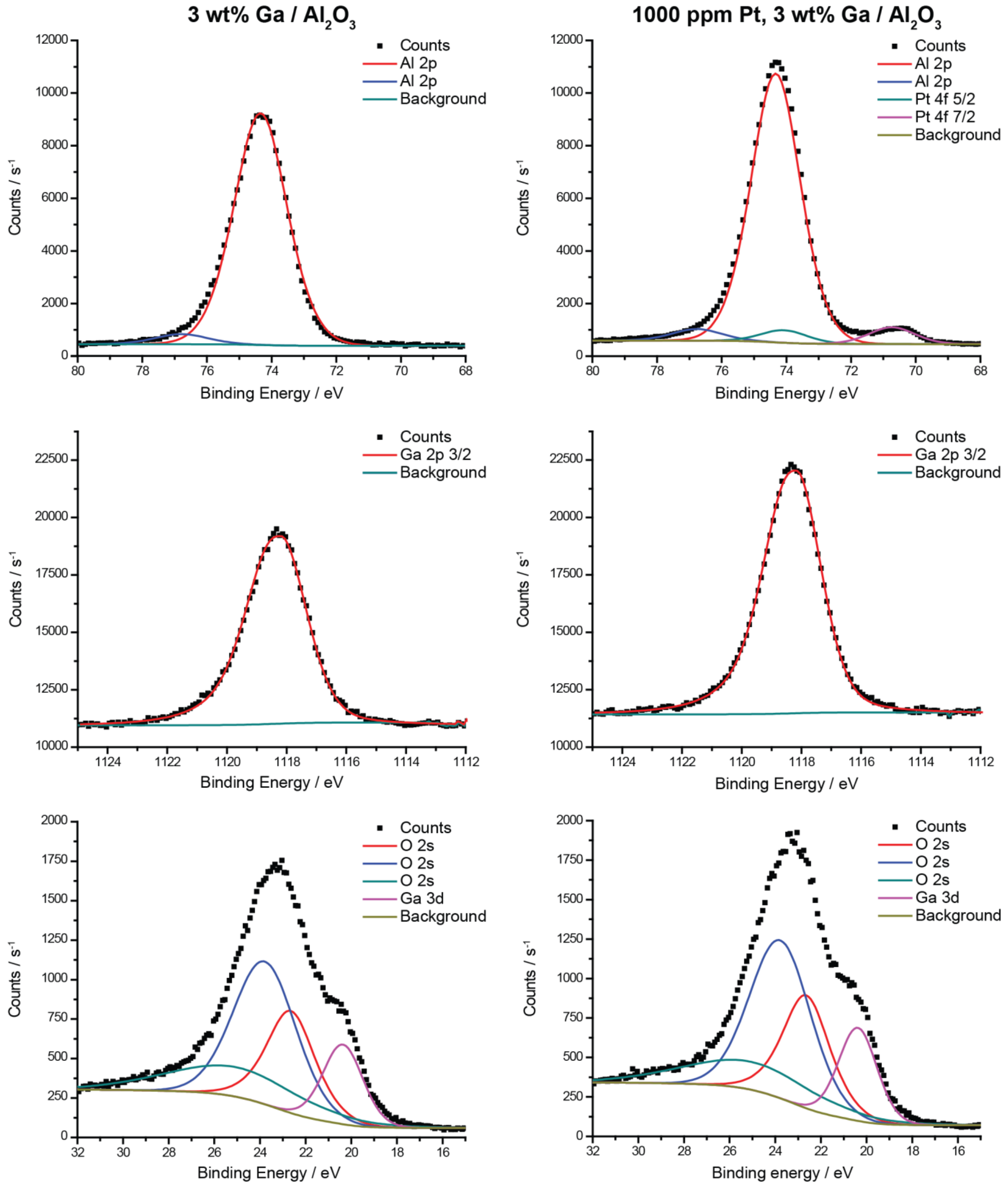

Figure S7: XPS spectra of the Al 2p (which includes Pt 4f)(top Figures), Ga 2p (middle Figures) and Ga 3d edges (bottom Figures), on the fresh 3Ga (left) and Pt3Ga (right) catalysts.

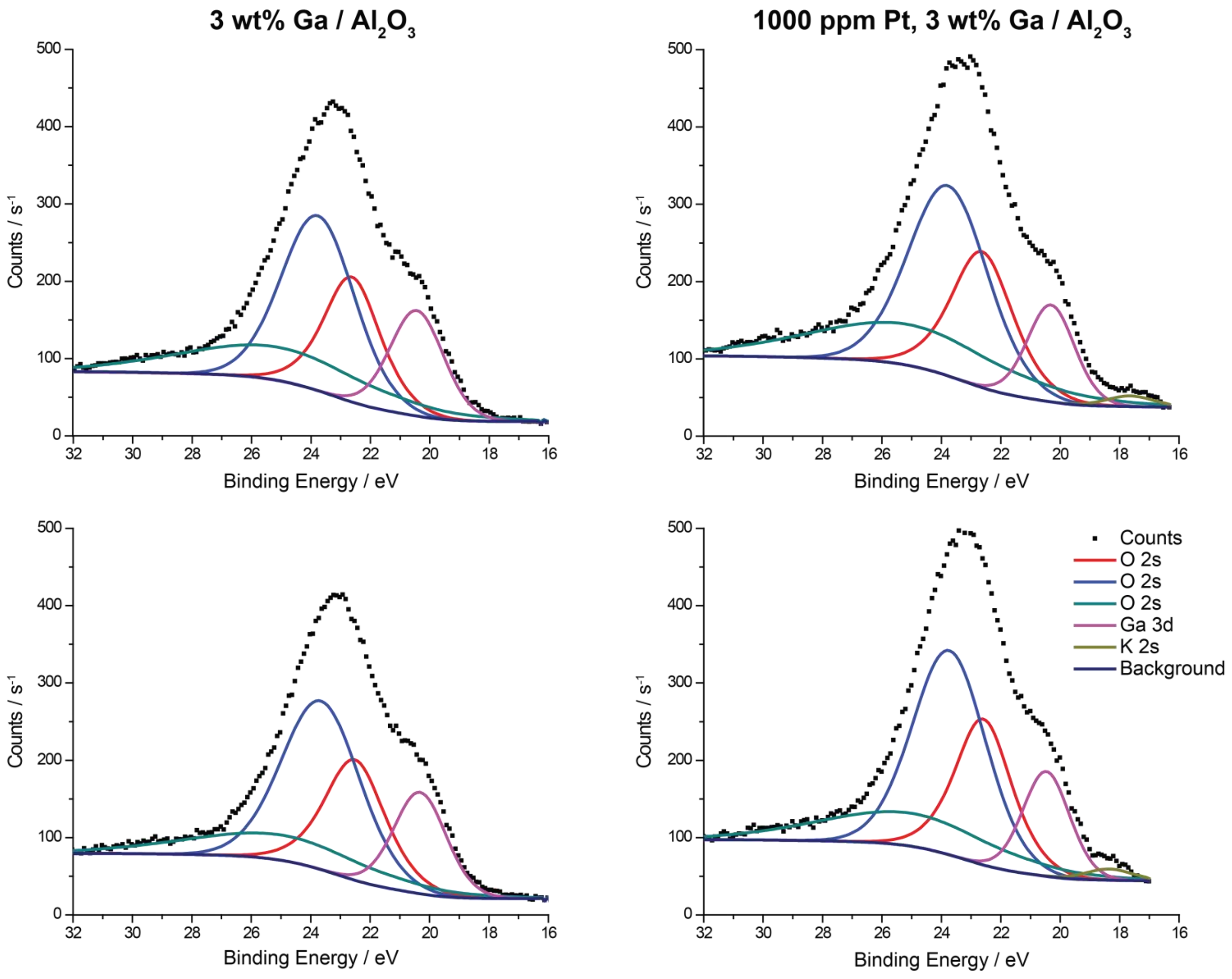

Figure S9: XPS spectra of 3Ga (left) and Pt3GaK (right) catalysts before (top) and after reduction (bottom) in an hydrogen atmosphere at 620ºC for 30 min. No evidence of the reduction of Ga is observed.
